# Recent Alterations in the Insurance Bill

**Published:** 1911-08-05

**Authors:** 


### RECENT ALTERATIONS IN THE INSURANCE BILL.

By a Correspondent.

The last development which is promised with regard to the National Insurance Bill may commend it to the Labour party, but hardly to the mass of the people. The British Medical Association is strenuous in opposing the regulation that the benefit of contract medical attendance should be given to all whose incomes escape the £160 a year limit. It considers that the benefit should be limited to those whose income from all sources does not exceed £2 a week. Mr. Lloyd George declines to omit the clause which grants medical treatment at the higher limit, but he is prepared to modify it. That is, he is willing that only artisans whose incomes exceed £2 a week should get their doctoring at the cheaper rate! This means that the clerk, the curate, the small tradesman, whose income is under £160 a year, will have to pay full fees, while the artisan who earns £3 a week will get the benefit of the cheaper treatment arranged for in the Bill. Yet, as we all know, the artisan has no external compulsion to spend his money on anything but his own comfort and that of his family, while the clerk must wear clothes of a certain stamp and live in a locality with at least a decent name! The curate—poor soul!—is expected not merely to fulfil all these conventions, but also to give of his scanty income in charity; and if he fails to do so his best parishioners will say reproachfully that he ought not to have gone into the Church unless he had private means. And the tradesman, even if his balance shows a debit, must, as a mere matter of advertisement, keep up a good appearance—dress his wife and daughter smartly, look flourishing, and even contribute his quota to local charities. Only a guaranteed rich man can afford to be mean in these particulars! It is mere playing to the gallery to admit certain classes to the benefits of the Bill and not all. If income is to decide fitness for benefits, let it be so, but let it be income alone, not income plus corduroys. The serge and the broadcloth are often thinner and more frayed and deserve the same consideration, if consideration for poverty is to be shown.

Happily, Mr. Lloyd George is prepared to give some consideration to hospital employees, who will, in case of illness, be treated within the walls of the establishment. "I am considering," he said, when interviewed by a representative deputation of hospital authorities on July 25, "whether in certain cases the sick pay of a person who is a charge upon a hospital shall not, in some form or another, be subscribed to the hospital itself. . . . Where a hospital undertakes to pay wages to a nurse during the time she is ill—within a limit—and where, in addition, it is prepared to give her medical attendance, the Government are prepared to reduce the charge by the amount of the sick pay. That, I think, will be a reduction of twopence a week." Twopence a week is not a despicable sum when employees are counted by hundreds, and, as Mr. Lloyd George explained, it was necessary that these should be insured under the Act, lest, if they fell ill in old age, they should have to fall back upon Poor Law aid.

Mr. Lloyd George was evidently willing that hospital authorities should appeal to Friendly Societies for some reimbursement of the cost of members treated in hospitals, but his suggestion: "You can in future say to a Friendly Society: 'If you go on sending patients to us—'!" was stopped short by the Hon. Sydney Holland's exclamation (which contained the sum of years of experience): "My dear sir, I have said it hundreds of times to Guardians without result." It is doubtful if Friendly Societies will prove more amenable than Boards of Guardians to the argument that they should pay the hospitals for the treatment their beneficiaries receive. The probabilities are, indeed, all the other way, and if the hospitals are not to be imposed upon they will need legislative support.

The pharmacists also are anxious, and honestly so, about the Bill. Contract drugging is apt to be cheap drugging, and there seems to be but little security that the drugs supplied to contract patients will be quite up to standard. The days may be gone when quassia, by reason of its bitter taste, could be passed off as quinine to the ignorant, nor do we want such methods revived. Yet it is, as Mr. W. F. Wells said at the meeting of the British Pharmaceutical Conference at Portsmouth, by no means certain that damaged drugs are sent abroad, and that of those which were not used elsewhere a considerable quantity returned, cooked or redressed in some way, to be consumed in this country. For this practice he blamed Guardians and governors of institutions, who insisted on having drugs at contract prices which could not be remunerative if the wares supplied were up to standard. Mr. Wells described this as a cruel and even a criminal thing, and with this verdict everyone must agree. But unless the sale of medicines is limited to apothecaries licensed by the State, and the State secures some guarantee of the quality of the medicines dispensed, contract supply may mean bad drugs. And if the State undertakes the task of supplying medicines, the question will arise whether the price promised is remunerative.

